# Shoulder movement complexity in the aging shoulder: A cross‐sectional analysis and reliability assessment

**DOI:** 10.1002/jor.24932

**Published:** 2020-12-08

**Authors:** Celeste L. Overbeek, Timon H. Geurkink, Fleur A. de Groot, Ilse Klop, Jochem Nagels, Rob G. H. H. Nelissen, Jurriaan H. de Groot

**Affiliations:** ^1^ Department of Orthopaedics Leiden University Medical Center Leiden The Netherlands; ^2^ Laboratory for Kinematics and Neuromechanics, Department of Orthopaedics and Rehabilitation Leiden University Medical Center Leiden The Netherlands

**Keywords:** aging, approximate entropy, motor control, physiotherapy, reliability, shoulder pathology

## Abstract

Healthy individuals perform a task such as hitting the head of a nail with an infinite coordination spectrum. This motor redundancy is healthy and allows for learning through exploration and uniform load distribution across muscles. Assessing movement complexity within repetitive movement trajectories may provide insight into the available motor redundancy during aging. We quantified complexity of repetitive arm elevation trajectories in the aging shoulder and assessed test–retest reliability of this quantification. In a cross‐sectional study using 3D‐electromagnetic tracking, 120 asymptomatic subjects, aged between 18 and 70 years performed repetitive abduction and forward/anteflexion movements. Movement complexity was calculated using the Approximate Entropy (ApEn‐value): [0,2], where lower values indicate reduced complexity. Thirty‐three participants performed the protocol twice, to determine reliability (intraclass correlation coefficient [ICC]). The association between age and ApEn was corrected for task characteristics (e.g., sample length) with multiple linear regression analysis. Reproducibility was determined using scatter plots and ICC's. Higher age was associated with lower ApEn‐values during abduction (unstandardized estimate: −0.003/year; 95% confidence interval: [−0.005; −0.002]; *p* < .001). ICC's revealed poor to good reliability depending on differences in sample length between repeated measurements. The results may imply more stereotype movement during abduction in the ageing shoulder, making this movement prone to the development of shoulder complaints. Future studies may investigate the pathophysiology and clinical course of shoulder complaints by assessment of movement complexity. To this end, the ApEn‐value calculated over repetitive movement trajectories may be used, although biasing factors such as sample length should be taken into account.

## 1 INTRODUCTION

In the upper limb, disorders that develop during aging, like rotator cuff pathology, are very common.^1^ The pathophysiology of these disorders is considered multifactorial and due to cascading events such as degeneration and overuse, but the true cause for shoulder region complaints is still not understood.^2^, ^3^, ^4^ We theorize that at some point in the degenerative process, people may be no longer able to find effective movement strategies, eventually leading to complaints.^2^, ^5^

The young and healthy human body has a redundant number of ways to execute a specific task, enabling learning through trial and error, quick adaptation to change, and uniform distribution of load across contractile tissues.^5^, ^6^, ^7^, ^8^ The complexity of repetitive movement trajectories (e.g., gait) has been interpreted as a characteristic of this motor redundancy, and thereby the healthiness of the underlying motor system.^9^, ^10^, ^11^, ^12^ A decreased complexity of movement during aging may suggest a person to move in a rigid and predictable way as the result of muscular and sensory degeneration and be the cause for slow decline in functioning and frailty.^8^, ^13^

In the shoulder, there is a marked degeneration of predominantly rotator cuff muscles during aging, requiring adaptation of the motor system to accomplish a task using different less affected muscles.^3^, ^4^ If motor redundancy becomes critical, this may predispose to the development of symptomatic disorders in the shoulder. The primary aim of this study was to determine shoulder movement complexity during abduction and anteflexion in 120 asymptomatic participants between 18 and 70 years old, to provide insight into the available motor redundancy during aging. Since the measurement of movement complexity in the shoulder is still in its infancy, we also performed a comprehensive reliability assessment as a base for future studies.

## 2 PARTICIPANTS AND METHODS

This was a Level II prognostic study in which the complexity of repetitive arm elevation trajectories in the aging shoulder was quantified and the test‐retest reliability of this quantification was determined.

### 2.1 Participants

**Figure 1:**
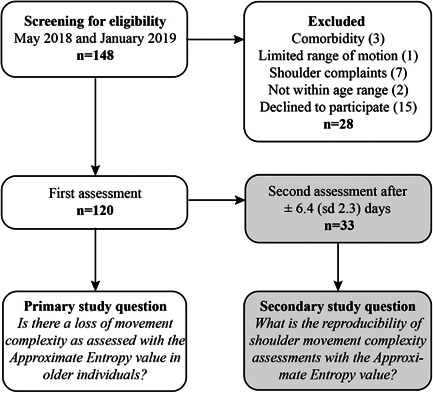
Flow diagram of participant enrolment

A prospective cohort study of asymptomatic participants, aged between 18 and 70 years was recruited through advertisements in public areas of the Leiden University Medical Center and via word‐of‐mouth between May 2018 and January 2019 (Figure 1). Exclusion criteria were previous shoulder complaints that lasted longer than a week or for which a general practitioner was consulted, previous shoulder fractures, previous shoulder surgery, tumors in the breast or shoulder region, radiation therapy in the shoulder region (including breast), no full range of motion, electronic implants, pregnancy or insufficient Dutch language skills. Eligible participants were analyzed at the laboratory of Kinematics and Neuromechanics (Leiden University Medical Centre, Leiden, The Netherlands). The study was approved by the Medical Research Ethics Committee. Participation was voluntary and all participants gave informed consent.

### 2.2 Measurement set‐up

The measurements were performed using a 3D‐electromagnetic movement registration system with a sampling frequency of approximately 17 Hz (Flock of Birds [FoB]; Ascension Technology Inc). This validated device is frequently used in shoulder motion measurement and can accurately (margin of error of 2°) determine the position of both arms in space.^14^, ^15^

During all measurements, participants were seated in the FoB with their trunk upright. According to instructions on a computer monitor, the investigator placed seven sensors in a standardized way. One sensor was placed on the skin overlying the manubrium sterni with Fixomull self‐adhesive bandage (Beiersdorf AG). Two sensors were adhered to the flat surface of the acromion just cranially to the acromial angle. Finally, using Velcro straps (Velcro Ltd), bilateral humeral and forearm sensors were fastened around the distal part of the humerus and distal part of the forearm, respectively. Subsequently, 24 bony landmarks were palpated by the investigator, registered with an eighth sensor and digitized to construct a patient‐specific 3D bone model relative to the seven sensors.^14^

The starting position was determined by asking the participant to sit up straight, with both arms in neutral position along the body. The participants were then asked to perform two movements, that is, maximum abduction and maximum anteflexion, with either of both arms (chosen by flipping a coin), at a comfortable speed with the thumb up. Each movement was repeated five times during each registration. A subgroup of 33 volunteers repeated the procedure after approximately 1 week, to determine the test–retest reliability.

### 2.3 Signal processing

**Figure 2:**
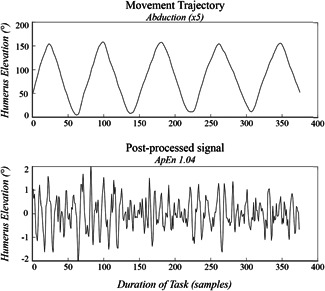
Example of abduction movement trajectories. Example of the humerus elevation trajectory during abduction (degrees) before (upper panel) and after 1.25 Hz High‐Pass filtering (lower panel)

The angular position of the humerus with respect to the thorax (thus capturing shoulder dynamics) was used for the analysis.^11^, ^16^, ^17^ The angular humerus elevation data vector per individual arm used for the analysis started from humerus first exceeding an elevation of 50° and ended when the humerus finally passed below 50° humerus elevation (Figure 2). The kinematic data was high‐pass filtered at a frequency of 1.25 Hz to filter the “static” components of movement control with custom‐made MATLAB software as depicted in Figure 2 (2018b release; The MathWorks).

### 2.4 Outcome measures

For the assessment of movement complexity, the Approximate Entropy value (ApEn‐value) was calculated using the function “ApproximateEntropy.m” in Matlab (2018b release; The MathWorks). The formula has been carefully described by Bruhn and coworkers.^18^ The ApEn‐value has been used in a wide range of pathologies and describes whether a system operates in a predictive, stereotype way or in a more chaotic, dynamic way, using many degrees of freedom.^5^, ^7^ Conceptually, the ApEn‐value describes the logarithmic likelihood that a repetition of *m* consecutive data points, will not be followed by another (*m* + 1) repeating data point.^18^, ^19^ The ApEn‐value ranges between 0 and (about) 2, where lower values represent great regularity in data (e.g., in a sine wave), whereas values close to 2 represent irregular complex data structures (e.g., gaussian noise).^20^ In accordance with the literature, *m* (the number of samples to be matched) was set at 2 and *r* (the criterium for assessing whether two samples are a match) at 0.2 *SD*.^19^, ^21^ As the ApEn‐value may depend on the data‐length and arm dominance, we controlled for this in our statistical analysis.^21^ The plane of elevation (degrees) and the maximum elevation height (degrees) were included in the statistical model to test whether these had an influence on the ApEn‐value.

### 2.5 Statistical analysis

Version 23 of the Statistical package of social sciences (SPSS, IBM Corp) was used for statistical analysis. Subjects were distributed in four age categories (18–70) years of age. The normality of data distribution was checked with histograms. Baseline characteristics were described with numbers and percentages, means, and 95% confidence intervals (95% CI) or *SD*, as appropriate.

The association between independent variable age and dependent variable ApEn‐value was analyzed by means of multiple linear regression analysis using a block‐enter method with controlling for task characteristics (duration of the task, plane of elevation, and maximal elevation), gender, and assessment of the dominant arm or not. The first block included age, gender, dominant side assessed, maximal elevation, the plane of elevation, and a linear factor of the task duration. In subsequent blocks, it was tested whether entering a quadratic and cubic form of the task duration resulted in a significant greater explanation of variance in ApEn‐value as based on an *R* ^2^ change of less than .10. Results of the regression analyses were presented using the standardized and unstandardized regression estimates with CIs and *p* values. A *p* value of less than .05 was considered to be statistically significant.

Reproducibility of the ApEn‐value was depicted in scatter plots and quantified using the average measures intraclass correlation coefficient (ICC), calculated in a two‐way mixed model with absolute agreement.^22^ To interpret the degree of reliability, the categorization by Cicchetti et al.^23^ was used: 0.00–0.40—poor agreement; 0.40–0.59—fair agreement; 0.60–0.74—good agreement; 0.75–1.0—excellent agreement.

### 2.6 Power analysis

Based on a power of 95% and an *α* of .05, it was anticipated that 111 participants would be required for an effect size of 0.3 with regression analysis (G*Power version 3.0.10). Accounting for approximately 10% loss of data, 120 participants were recruited. For reliability analyses, it is advised to at least recruit 30 participants.^24^ Again, to account for 10% loss of data, 33 out of 120 participants (28%), performed a second assessment.

## 3 RESULTS

Baseline characteristics are described in Table 1.

**Table 1:** Participant characteristics

	Asymptomatic participants *n* = 120	
Demographics		
Age, year (mean, *SD*)	43.6 (14.9)	
Female (*n*, %)	67 (56)	
Right side dominance (*n*, %)	110 (92)	
Dominant side assessed (*n*, %)	60 (50)	
BMI (mean, *SD*)	24.0 (3.7)	
Profession (*n*, %)		
Unemployed (*n*, %)	12 (10)	
Seated (*n*, %)	99 (82.5)	
With upper limb activity above head (*n*, %)	9 (7.5)	
Sports		
No sports (*n*, %)	15 (12.5)	
Sports with upper limb activity below head (*n*, %)	55 (44.2)	
Sports with upper limb activity above head (*n*, %)	52 (43.3)	
Hours/week	3.8 (2.8)	
Clinical score		
Self reported general health	18 (12–29)	
Excellent (*n*, %)	31 (25.8)	
Very good (*n*,%)	49 (40.8)	
Good (*n*, %)	39 (32.5)	
Fair (*n*, %)	1 (0.8)	
Bad (*n*, %)	0 (0)	
Constant Shoulder score dominant arm (median, qrtls)	96 (93; 100)	
Constant Shoulder score nondominant arm (median, qrtls)	95 (92; 100)	
VAS for pain in rest (median, qrtls)	0 (0; 3)	
VAS for pain during movement (median, qrtls)	1 (0; 3)	
VAS for daily functioning (median, qrtls)	0 (0; 3)	
Measurement characteristics		
Assessment of dominant arm		
18–31 Years (*n*, %)	17 (50)	*χ* ^2^: 0.501
32–45 Years (*n*, %)	14 (47)	*p* Value: .919
46–58 Years (*n*, %)	14 (48)	
59–70 Years (*n*, %)	15 (56)	
Samples during abduction (*n*, *SD*)		
18–31 Years (*n*, *SD*)	308 (113)	F‐statistic: 1.566
32–45 Years (*n*, *SD*)	271 (99)	*p* Value: .201
46–58 Years (*n*, *SD*)	263 (72)	
59–70 Years (*n*, *SD*)	271 (66)	
Samples during anteflexion (*n*, *SD*)		
18–31 Years (*n*, *SD*)	311 (120)	F‐statistic: 0.045
32–45 Years (*n*, *SD*)	309 (135)	*p* Value: .987
46–58 Years (*n*, *SD*)	302 (91)	
59–70 Years (*n*, *SD*)	303 (83)	

Abbreviations: BMI, body mass index; N, number; *SD*, standard deviation; VAS, Visual Analogue Scale.

**Figure 3:**
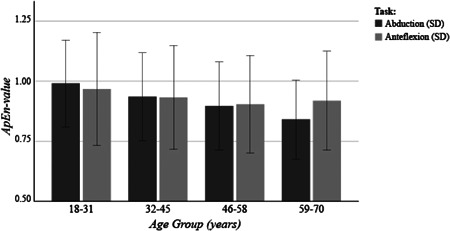
Bar chart of ApEn values during abduction and anteflexion. *SD*, standard deviation

For abduction, the ApEn‐value declined from 1.01 (*SD*: 0.16), in the age‐category of 18–31 years old, to 0.84 (*SD*: 0.16), in the age‐category of 59–70 years old (Figure 3). Accordingly, higher age was associated with lower ApEn values (estimate: −0.003 per year, 95% CI [−0.005; −0.002], *p* < .001) during the abduction task (Table 2). The only factor further associated with the ApEn‐value was sample length (Table 2).

**Table 2:** Approximate Entropy value in asymptomatic participants as predicted by age and potential covariates for the repeated abduction and anteflexion movements

	Approximate Entropy value				
	Standardized coefficient	Unstandardized coefficient	95% CI with unstandardized coefficient	*p* Value	Adj. *R* ^2^
Abduction					
Intercept		0.449	[0.038; 0.860]	NA	.651
Age	−0.252	−0.003	[−0.005; −0.002]	**<.001**	
Sex*	0.101	0.037	[−0.003; 0.076]	.070	
Dominant side assesed**	−0.029	−0.011	[−0.051; 0.030]	.611	
Plane of elevation°	−0.019	0.000	[−0.003; 0.002]	.935	
Maximal elevation°	−0.005	0.000	[−0.003; 0.002]	.758	
Sample length (linear)	1.651	0.003	[0.002; 0.004]	**<.001**	
Sample length (quadratic)	−0.944	−2.9 × 10^−6^	[−5.0 × 10^−6^; −1.0 × 10^−6^]	**.001**	
Anteflexion					
Intercept		0.327	[−0.074; 0.728]	NA	.636
Age	−0.100	−0.001	[−0.003; 0.000]	.090	
Sex (female is ref.)	−0.009	−0.004	[−0.053; 0.045]	.870	
Dominant side assesed (no is ref.)	0.102	0.043	[−0.004; 0.091]	.072	
Plane of elevation°	0.033	−0.002	[−0.005; 0.000]	.101	
Maximal elevation°	−0.104	0.001	[−0.002; 0.003]	.587	
Sample length (linear)	1.527	0.003	[0.002; 0.004]	**<.001**	
Sample length (quadratic)	−0.748	−2.0 × 10^−6^	[−3.0 × 10^−6^; −8.0 × 10^−7^]	**.001**	

*Note*: Multivariate regression analysis. Significant values at the *α* = .05 in bold.

Abbreviation: CI, confidence interval.

* *Reference is female*.

** *Reference is nondominant side*.

For anteflexion, the ApEn‐value declined slightly from 0.98 (*SD*: 0.23) in the age category of 18–31 years old, to 0.94 (*SD*: 0.20) in the age category of 59–70 years old (Figure 3). Age was not associated with the ApEn value (estimate: −0.001 per year, 95% CI: [−0.003; 0.000], *p* = .090) during the anteflexion task (Table 2).

**Figure 4:**
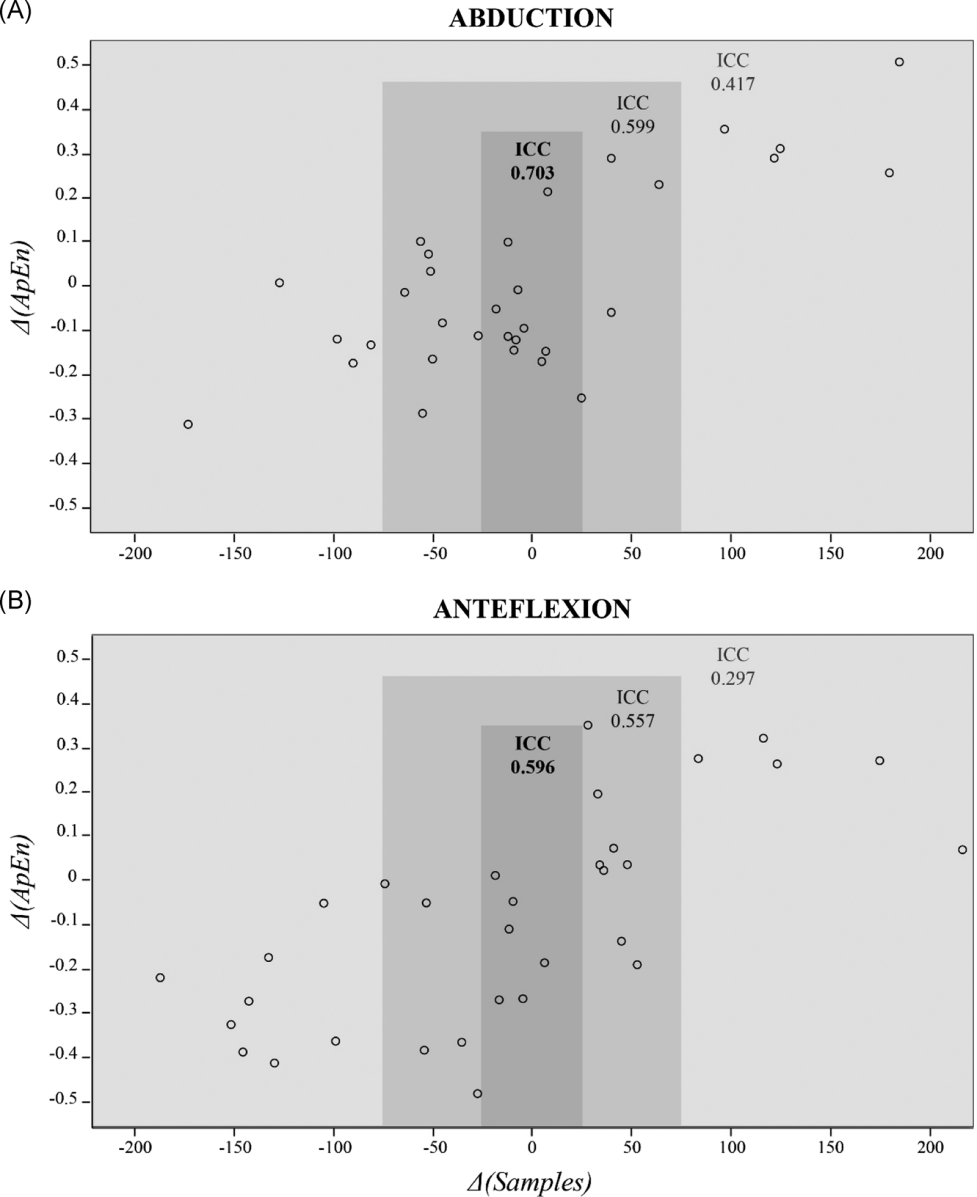
Reproducibility of assessment of motor complexity using the Approximate Entropy value. The difference in sample length between the first and second assessment Δ(Samples) is plotted against the difference in Approximate Entropy value between the first and second assessments Δ(ApEn). The reproducibility of the ApEn value was calculated with the intraclass correlation coefficient (ICC) for the data vectors differing less than 25 samples (abduction: *n* = 10, anteflexion: *n* = 6), less than 75 samples (abduction: *n* = 22, anteflexion: *n* = 19) and less than 200 samples (*n* = 33) between the first and second assessment

A total of 33 (28%) participants with a mean age of 48 years (*SD*: 14 years), 46% women, and right‐side dominance 94%, performed a second assessment after a mean of 6.4 days (*SD*: 2.3). The ICC's for the overall group were: 0.417 (95% CI: [0.084; 0.664]) during abduction, and 0.297 (95% CI: [−0.028; 0.571]) during forward flexion. We observed that the difference in ApEn‐values (ΔApEn) between both assessments strongly depended on the difference in duration (i.e., the number of samples) of the task between both assessments (ΔSamples). Figure 4 exemplifies the association between ApEn‐values of the first and second assessment and the difference in number of samples between those assessments. In the case of small differences in duration of the task between both assessments (i.e., ΔSamples < 25), the agreement was good (Figure 4).^23^

## 4 DISCUSSION

In the current study, we determined shoulder movement complexity during abduction and anteflexion in 120 asymptomatic participants between 18 and 70 years old, to provide insight into the available motor redundancy during aging. Since measurement of movement complexity in the shoulder is still in its infancy, we also performed a comprehensive reliability assessment as a base for future studies. In line with the common loss‐of‐complexity hypothesis, we found a significant age‐related decline in movement complexity during abduction, which may imply more stereotype movements and less ability to adapt to stresses during aging, making the movement prone for development of complaints.^5^, ^6^, ^7^, ^8^ Assessing the complexity of repetitive movement trajectories proved reliable, although severely dependent on the length of data.

The decline in movement complexity we found in older individuals during abduction may be due to a loss of functional components (e.g., muscle atrophy) and/or altered coupling between those components (e.g., central degeneration).^25^ Several factors could explain why the decline in movement complexity was present during abduction and not during anteflexion. In contrast to the abduction task, the movement trajectory of the anteflexion task is nearly completely within the visual field, which may allow for compensation of functional loss.^26^ In addition, participants might be more skilled in the execution of anteflexion rather than abduction tasks as fine motor skills are most commonly performed in front of the body.^27^, ^28^, ^29^ It could be that during “less‐challenging” tasks, movement complexity might be similar in elderly and young people, and that more “challenging” tasks are required to detect changes in movement complexity during aging.^5^, ^9^, ^11^

Age‐associated decline in complexity of motor output has been observed in various regions of the musculoskeletal system, including gait and postural control.^30^, ^31^, ^32^ Reduced movement complexity in walking patterns of elderly has been associated with the risk of frailty and a consequent risk for falling.^33^, ^34^, ^35^ Furthermore, individuals who have to make repetitive movements with little variability (e.g., wheelchair users, assembly line workers, butchers) have been shown to be more likely of developing overuse disorders when they have reduced movement complexity on beforehand.^16^, ^28^, ^29^, ^36^, ^37^, ^38^, ^39^ For that matter, movement complexity may be an interesting and easy to access prognostic factor for shoulder pathologies.^16^, ^33^, ^34^, ^35^, ^36^, ^37^, ^40^, ^41^, ^42^

The prospective design and relatively large number of participants were strong points of our study, but some limitations should be acknowledged. First, we cannot rule out the presence of a selection bias due to the fact that participants were recruited via advertisements, which may result in inclusion of participants with a specific interest for shoulder (dis‐)functioning. However, since the outcome of interest is an objective measure, we do consider it unlikely that selection bias hampered generalizability in this study. Second, our outcome measure, the ApEn‐value has not been extensively validated in the assessment of shoulder movement complexity.^21^ Hampering the comparability of our data, we found a strong association between the ApEn‐value and the duration of the motor task, although this did not affect conclusions regarding the association between age and ApEn‐values, since the distribution of sample length was equal across the age‐groups and controlled for in the regression analyses. Thirdly, we included participants based on a clinical assessment and did not rule out asymptomatic pathologies through radiological examination. Hence, participants with asymptomatic shoulder pathology may have been included in this study. It has been previously shown that reduction in movement complexity is multifactorial and between‐patient variance in movement complexity exists in the presence of comparable local pathology.^42^, ^43^ Therefore, while participants with asymptomatic shoulder pathology may have been included in this study, we do not think that this affects the possible clinical implication of our finding that there is reduced movement complexity during abduction in elderly, which may indicate vulnerability to developing complaints. Fourthly, we performed the measurements during only abduction (*p* < .001) and anteflexion (*p* = .09) tasks and therefore, we cannot conclude whether a loss of movement complexity during aging is isolated or diffuse. On itself, the finding of reduced movement complexity manifesting predominantly during abduction is interesting considering the fact that shoulder pathology is associated with this movement.^44^ However, in future assessments it may be interesting to investigate whether the reduction in movement complexity is indeed isolated to the abduction movement (and possibly asymptomatic pathology) or diffuse by assessing movement complexity during other movements, for example, axial humeral rotation. Finally, while this study was initiated to provide a base for research in symptomatic patients, findings related to reduction of movement complexity during abduction in elderly, as well as findings related to reliability, may not be extrapolatable to symptomatic patients.

Shoulder complaints are highly prevalent in western societies and have a great impact on an individual's ability to perform daily activities and quality of life.^44^, ^45^, ^46^ Currently, the pathophysiology of common shoulder complaints is not clear, but there is increasing evidence that behavioral/dynamic factors play a crucial role.^2^, ^47^, ^48^ We theorize that movement complexity may contribute to whether one is able to maintain symptomless function in case of functional decline and stress on (contractile) tissues in the shoulder.^45^ To further study this theory, we suggest to quantify shoulder movement complexity.^43^

We designed a method for measuring movement complexity in the shoulder, applying criteria (*m* and *r*) for the calculation of the ApEn‐value in accordance with the literature, to enhance statistical reproducibility and comparability.^22^ As has been described earlier, we found a strong association between the ApEn‐value and the duration of the motor task.^21^ This became clear during the main regression analysis, but even more so in the reliability assessment. Therefore, duration of motor task recording has to be taken into account when assessing shoulder movement complexity.^21^ In future studies, this problem can be avoided by extending measurement time up to the level where the ApEn‐value reaches a plateau phase (in our case >400 samples).^21^

In this prospective cross‐sectional cohort study with assessment of shoulder movement complexity in 120 participants between the age of 18 and 70 years old, we found that higher age was associated with a decline in movement complexity during abduction, indicating reduced motor redundancy during this movement. If the redundancy of ways to execute a specific task becomes critical, adapting to change and distributing load equally across tissues may become difficult.^5^, ^6^, ^7^, ^8^ Therefore, our finding of reduced motor redundancy during abduction in older individuals could play a role in the frequent onset of abduction‐related shoulder (overuse) complaints in this population.^44^, ^45^, ^46^ In future studies, movement complexity may be assessed to study the pathophysiology and clinical course of shoulder complaints. To this end, the Approximate Entropy value calculated over repetitive movement trajectories may be used, although biasing factors such as data length should be taken into account.

## AUTHOR CONTRIBUTIONS

All authors had substantial contributions to the research design, acquisition, analysis and/or interpretation of data; drafting the paper and/or revising it critically; approval of the submitted and final versions.
